# Inkjet-Printed Localized Surface Plasmon Resonance Subpixel Gas Sensor Array for Enhanced Identification and Visualization of Gas Spatial Distributions from Multiple Odor Sources

**DOI:** 10.3390/s24206731

**Published:** 2024-10-19

**Authors:** Tianshu Jiang, Hao Guo, Lingpu Ge, Fumihiro Sassa, Kenshi Hayashi

**Affiliations:** Department of Electronics, Graduate School of Information Science and Electrical Engineering, Kyushu University, Fukuoka 819-0395, Japan; jiang.tianshu.031@s.kyushu-u.ac.jp (T.J.); guo.hao.097@s.kyushu-u.ac.jp (H.G.); gu.lingpu.755@m.kyushu-u.ac.jp (L.G.); sassa@ed.kyushu-u.ac.jp (F.S.)

**Keywords:** inkjet-printed LSPR gas sensor array, localized surface plasmon resonance, inkjet printing, gas identification, gas spatial distribution, subpixel pattern, Au/Ag nanoparticle, fluorescent pigment, chemical imaging

## Abstract

The visualization of the spatial distributions of gases from various sources is essential to understanding the composition, localization, and behavior of these gases. In this study, an inkjet-printed localized surface plasmon resonance (LSPR) subpixel gas sensor array was developed to visualize the spatial distributions of gases and to differentiate between acetic acid, geraniol, pentadecane, and cis-jasmone. The sensor array, which integrates gold nanoparticles (AuNPs), silver nanoparticles (AgNPs), and fluorescent pigments, was positioned 3 cm above the gas source. Hyperspectral imaging was used to capture the LSPR spectra across the sensor array, and these spectra were then used to construct gas information matrices. Principal component analysis (PCA) enabled effective classification of the gases and localization of their sources based on observed spectral differences. Heat maps that visualized the gas concentrations were generated using the mean squared error (MSE) between the sensor responses and reference spectra. The array identified and visualized the four gas sources successfully, thus demonstrating its potential for gas localization and detection applications. The study highlights a straightforward, cost-effective approach to gas sensing and visualization, and in future work, we intend to refine the sensor fabrication process and enhance the detection of complex gas mixtures.

## 1. Introduction

Detection of volatile organic compounds (VOCs) is important for a broad range of applications [1,2,3], including environmental monitoring [4], biotechnology [5], food safety [6], adulteration detection [7,8], and healthcare [9,10,11]. Measurement of the spatial distributions of VOC gases can provide valuable information for use in both modern industrial production, e.g., for toxic gas detection [12,13], and daily life, e.g., in screening for spoiled food [14,15]. Additionally, by analyzing the way in which these gases disperse in the presence of airflow, robots equipped with advanced recognition algorithms can be used to pinpoint the location of odor sources to identify potential threats or simply make our daily lives more convenient [16,17,18]. Moreover, examining the spatial distributions of gases as they emanate from odor sources will also provide a deeper understanding of the properties of the gas source, including its composition, location, and temporal variations [19,20,21,22].

The gathering of this type of gas spatial information can be realized by using gas concentration information acquired from the different gas sensors in a sensor array from various locations and applying different detection methods. Metal oxide semiconductor (MOS) gas sensors, which use materials such as SnO_2_ [23], SiC [24], Ga_2_O_3_ [25], and ZnO [26], are widely used iFn gas detection applications. The changes in the resistance rates of these metal and semiconductor materials under different gas environments can be used to detect the gas concentration. Bai et al. used SnO_2_ nanoflakes grown on SnO_2_ nanotubes to fabricate a new nanocomposite for use in gas sensing and achieved detection of NO_2_ at concentrations of less than 10 parts per billion (ppb) with relatively long-term stability [23]. Although MOS sensors are characterized by high sensitivity and fast response times, they still face challenges in practical applications, including low selectivity, high operating temperatures, and susceptibility to aging [27]. Quartz crystal microbalance (QCM) sensors can detect the existence and concentrations of gases through changes in the quartz crystal resonant frequency caused by gas mass changes on the quartz crystal. Because quartz shows low gas absorption properties, different types of sensing material coatings must be used, e.g., molecularly imprinted sol–gel (MISG) [28], metal–organic frameworks (MOFs) [29], and carbon nanotubes [30]. Liu et al. reported QCM gas sensors with MISG coatings in a sensor array to distinguish volatile aldehyde vapors that showed selectivity in the presence of hexanoic acid, nonanoic acid, and benzoic acid. QCM gas sensors offer high sensitivity, rapid response times, and room temperature operation capabilities. However, the selectivity of QCM sensors is heavily reliant on the use of high-performance sensing materials and is affected significantly by environmental factors such as humidity, temperature, and pressure during operation [28]. Electrochemical sensors can provide rapid responses, high selectivity, and high sensitivity, and operate by measuring the current changes that occur when the electrolyte coating on the working electrode undergoes an oxidation or reduction reaction on contact with the target gas. Sreerag et al. enhanced the accuracy of carbon dioxide detection by using ionic liquids and achieved a limit of detection (LOD) of 0.53 ppm [31]. Electrochemical sensors are noted for their selectivity and rapid responses; however, their electrodes and electrolytes suffer from relatively short lifespans and are highly susceptible to environmental influences. Recently, localized surface plasmon resonance (LSPR) has emerged as an innovative approach to providing an efficient gas-sensing technique. Changes in the absorption spectrum caused by the LSPR effect are primarily influenced by the type of gas to be detected, the characteristics of the metal nanoparticles used, and the components that affect the electric field around the metal nanoparticles, e.g., fluorescent pigments [32]. For a single gas, the spectral changes are mainly associated with the different metal nanoparticles and fluorescent pigments, which can serve as a fingerprint for the gas to allow specific gas molecules to be identified and distinguished, thereby providing selective gas detection. Additionally, LSPR gas sensors offer high sensitivity and rapid responses that enable real-time detection of different gas types and concentrations, thus making them suitable for applications that require rapid responses, e.g., gas-tracking robots [16].

In this work, we have developed a 2D LSPR sensor array that is fabricated by inkjet printing and is capable of distinguishing different gases while also capturing their spatial distributions. Acetic acid, geraniol, pentadecane, and cis-jasmone were selected as target gases for detection. Each sensor comprises a detection unit composed of 16 subpixels, where these subpixels consist of combinations of different metal nanoparticles and pigments. The diverse changes in the absorption spectra across these subpixels enrich the descriptive information that can be acquired for the gas signatures. Using a hyperspectral camera, we recorded these LSPR spectral changes and their spatial positioning when caused by gas molecules. By sampling the spectra from the different detection units in each sensor and calculating the mean squared error (MSE) versus spectra that were obtained in our previous work [32,33], we quantified the changes in the gas concentrations. Furthermore, we used principal component analysis (PCA) to extract features from the spectral variations that occurred across the 16 subpixels of each sensor, thus enabling differentiation of the different gases. Subsequently, the MSE and PCA values were used to create heat maps by treating each sensor as a pixel. The MSE and PCA indices determined the depth of color and the hue of each pixel, respectively. This study introduces a new principle for achieving gas visualization and designs and fabricates a new detection system compared to previous work. In contrast to methods such as visualization using fluorescent dyes, this approach detects gas concentration and distinguishes the spatial distribution of different gas types [22]. Additionally, compared to surface-enhanced Raman scattering sensors visualization methods of our former work [19], this method is more convenient with a low response time and offers higher information density, aiding in differentiating gas components in complex environments. This gas-sensing approach is highly promising for visualization of the spatial distributions of VOC gases, thus making it valuable for applications including odor source localization, environmental pollution monitoring, and enhancement of healthcare outcomes.

## 2. Related Work

The visualization of the spatial distributions of gases in detection systems generally depends on specific prerequisites. First, detection systems often use multi-channel sensors or sensor arrays: when they measure the gas concentrations and types, the sensors in the different channels can also record the spatial positioning of the gas signals based on their relative locations. Second, these sensors require high sensitivity and rapid response times: to represent the real-time spatial distributions of the gases accurately, the sensor units must reflect changes in the gas information at specific locations quickly to avoid discrepancies between the measured and actual gas distributions. Third, the relative spatial positioning of the sensors must be quantifiable and recordable: abstract gas information can only be projected onto the actual space by mapping the measurement space accurately, thus achieving the required transformation from real space to a visualized gas distribution.

In previous studies, QCM sensor-based visualization systems have been assembled for gas flow detection. For example, Ishida et al. developed a multichannel sensor array composed of 21 QCM sensors to track odor flow [34]. In this research, each sensor had dimensions of 4 mm × 8 mm, and adjacent sensors were spaced 1.27 cm apart in the array. Their portable and cost-effective system demonstrated an ability to visualize gas flows at speeds up to 30 cm/s. However, only the frequency change of the quartz could be passed on and used to perform the visualization, and the sensing material could only absorb the target gases, which can present difficulties when facing a complex gas environment or aiming to perform multi-gas detection.

Iitani et al. developed a fluorometric sensor system for visualization of VOC distributions [22]. A 90 mm × 90 mm mesh with immobilized alcohol dehydrogenase (ADH) was used to detect ethanol gas concentrations from transcutaneous measurements. When the ethanol gas interacted with the mesh, which was wetted using nicotinamide adenine dinucleotide (NAD), the NAD was reduced through the ADH-mediated reaction. The reduced NADH can emit fluorescence under ultraviolet (UV) light illumination, which then allows the ethanol gas distribution to be visualized by capturing the fluorescence intensity via a camera. This approach provided high-resolution images of the spatial distribution of the gas to be detected. However, fluorometric sensors require suitable fluorescent dyes that are specific to the target gas and must also account for potential interference from the environmental lighting conditions.

Tao et al. developed an innovative approach by using a colorimetric sensor array (CSA) that was integrated with chemometric methods to perform qualitative discrimination of maize silage [35]. They selected 12 color-sensitive dyes to create the CSA by functioning as artificial olfactory sensors to capture the odor fingerprints of the maize silage. Machine vision algorithms were then used to extract the color features, and PCA was applied to reduce the dimensionality of the data. The PCA results were then used as input variables to develop various qualitative discrimination models, including support vector machine (SVM), extreme learning machine (ELM), and random forest (RF) models, to realize the high recognition rates required. However, the CSA developed in this case could not realize a rapid response and the array showed low integration, with limited information being available for further analysis.

Like the other gas detection technologies described above, inkjet-printed subpixel LSPR sensors also face several pressing challenges. These challenges include requirements to (1) perform label-free discrimination of gases within a single area, (2) develop methods to fabricate high-density and low-cost integrated sensor arrays, and (3) correlate the gas information obtained with spatial and temporal distributions.

## 3. Materials and Methods

In this section, the fabrication method of the inkjet-printed subpixel gas sensor array and the gas detection process will be introduced. Additionally, we also describe the details required for visualization of the gas spatial distribution and compare the spectral information differences among the gases of interest.

### 3.1. Fabrication of Subpixel Gas Sensor Array

#### 3.1.1. Materials

The subpixel gas sensor is fabricated using a combination of gold nanoparticles (AuNPs), silver nanoparticles (AgNPs), and fluorescent pigments. The AuNPs (60 nm diameter, OD 1) and AgNPs (75 nm diameter), and polyvinyl pyrrolidone (PVP) with a molecular weight of 40,000 Da (K 30) were purchased from Sigma-Aldrich Chemical Co., Ltd, Tokyo, Japan. Triton-100 was purchased from Shin-Etsu Chemical Co., Ltd, Tokyo, Japan. Eosin Y, Eosin 10B geraniol, pentadecane, and acetic acid were purchased from Fujifilm Wako Co. Ltd, Tokyo, Japan. Cis-jasmone and rhodamine B were purchased from TCI Co. Ltd, Tokyo, Japan. Rhodamine 6G was purchased from Waldeck Co. Ltd, Münster, Germany. Polyethylene terephthalate (PET) transparent film was purchased from Plus Corporation. All reagents were used as received without any pretreatments.

#### 3.1.2. Fabrication of the Subpixel Pattern

**Figure 1:**
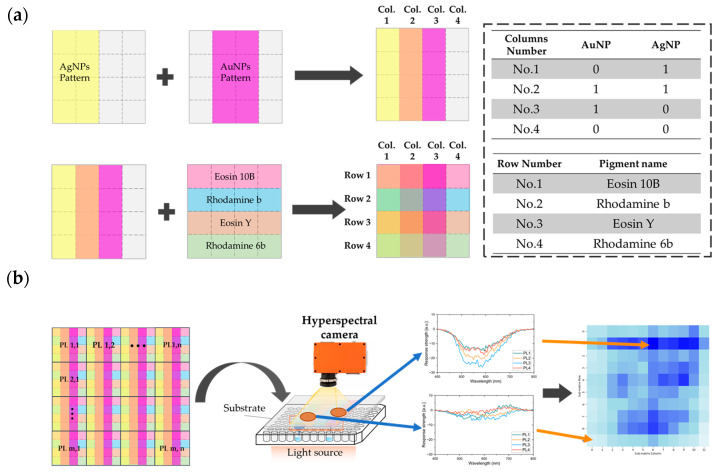
Fabrication process for the inkjet-printed subpixel gas sensor and its detection method. (**a**) Printing of silver nanoparticles (AgNPs; in yellow) and gold nanoparticles (AuNPs; in purple), which are arranged by column, and the pigments, which are arranged in rows. (**b**) Detection and visualization process of the inkjet-printed subpixel gas sensor. The wavelength figures here depict four sample subpixels for comparison and explanation.

The methods for fabrication of nanoparticle inks and formation of nanoparticle patterns on the PET film were reported in our previous work [32] and are also explained in the Supplementary Materials. As illustrated in Figure 1 and Figure S1, each sensor is composed of multiple subpixels with slide length is 3 mm, and the total slide length is 12 mm, which is limited by inkjet printer used in this experiment. The sensor array can be assembled in different scales, and in this experiment, we made the sensors into an 11 × 12 array to fulfill the hyperspectral camera’s field of view requirements for visualization.

The AgNPs and AuNPs were printed along the longitudinal direction in the first and second columns, and in the second and third columns of each sensor, respectively, to produce four different nanoparticle patterns. Then, in each row of the sensor, Eosin 10B, rhodamine B, Eosin Y, and rhodamine 6B were printed to create the subpixels, with each being formed by different combinations of the fluorescent pigments and the nanoparticles. The fabricated inkjet-printed subpixel sensor array is shown in Figure S2. As a result, the 16 subpixel sensors are placed in a 4 × 4 array to provide gas spectra information and form the sensor, and multiple sensors are then accumulated to form the inkjet-printed sensor array.

### 3.2. Detection of the Gas

The fabricated sensor array substrate was positioned at a height of 3 cm above the gas source to perform the experiment. The LSPR spectral measurement system is shown in Figure S3. The gas source consisted of circular blind holes arranged in an array, with each hole having a diameter of 7 mm and a depth of 6 mm. Here, the size of blinding holes and distance are determined according to our former research to get stable result, and the impact of these parameters on the experimental results will be discussed in our future work [36]. To form the gas source region, 200 µL of the gas solution to be evaporated was placed in each blind hole. According to our former research [16], the LSPR sensor is sensitive to humidity changes, which can also affect the gas evaporation rate. To minimize environmental influences, the experimental area is maintained at 25 °C with 60% humidity throughout this study. The spectral images of acetic acid, geraniol, pentadecane, and cis-jasmone were then gathered from the substrate using the hyperspectral camera at times of 0 (the start of the experiment), 1, 3, and 5 min. When a single gas was to be measured, the gas source was placed at the center of the hyperspectral camera’s field of view. For simultaneous monitoring of the spatial diffusion of two gases, the two gas sources were positioned at the lower left and at the upper right of the hyperspectral camera’s field of view. During detection, the gas molecules around the hot spots of the nanoparticles interact with incident light, generating distinct emission spectra, thereby enabling molecular desorption and gas detection. According to our previous research, the sensitivity of the LSPR gas sensors can reach 3.5 ppm, making it possible to visualize gases even in low-concentration environments [37].

Absorption spectra from the different spatial regions were analyzed using the spectral images acquired by the array. By calculating differential spectra between the spectral images at any given time and those acquired at the start of the experiment, LSPR spectra signals induced by gas molecules could be identified. Additionally, gas spectral information acquisition from each subpixel sensor was performed for each of the 16 subpixels, thus allowing for analysis of the spectral changes for the various combinations of the nanoparticles and the fluorescent pigments under the same conditions for the same gas.

Spectral data were also collected for the gas solution at different concentrations using the same method that was used for the single gas spatial diffusion measurements. In this study, the spatial distributions of the gas solutions with concentrations measured at 10%, 30%, 50%, and 100% of the original concentration were compared to find the differences between these solutions.

### 3.3. Visualization of Gas Spatial Distribution

**Figure 2:**
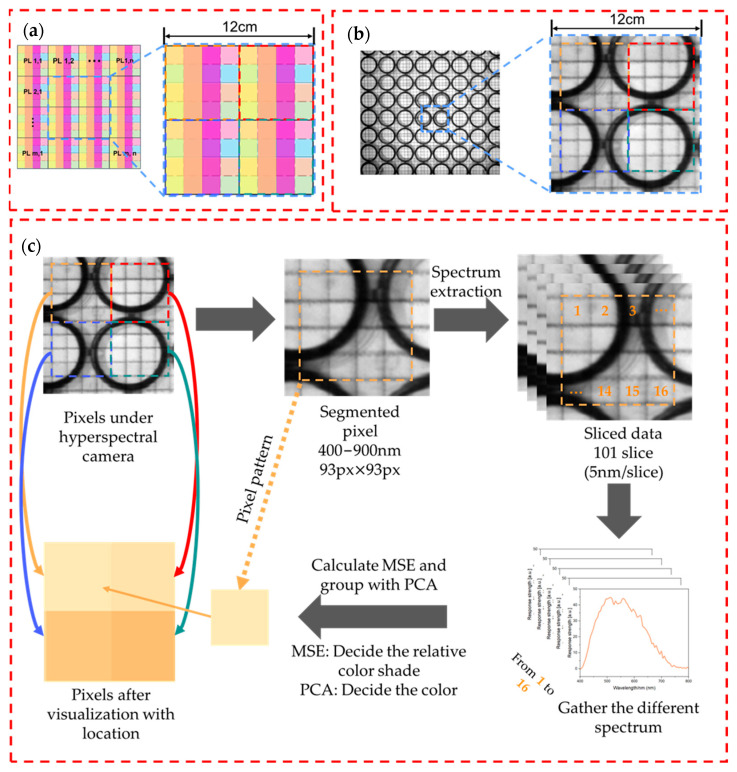
(**a**) Schematic of the inkjet-printed subpixel sensor array and (**b**) the spectral figure obtained from the hyperspectral camera with 4 subpixel sensors. (**c**) Digitalization and visualization process from spectral figure to heat map, where one pixel in the heat map represents a sensor with 4 × 4 subpixels.

For each sensor in the sensor array, the differential spectral change in response to the gas is inversely proportional to the distance between the sensor and the gas source. By analyzing the spectral changes of the sensors at the different positions within the array, the spatial distribution of the gas can then be determined across the entire sensor matrix area. Here, the hyperspectral camera was used to extract the spectral information for each pixel within its field of view. By comparing the absorption spectra acquired from each pixel at any given time with the initial absorption spectra, differential spectra corresponding to each pixel can be obtained, thus resulting in acquisition of differential images. As shown in Figure 2a, each sensor contains 16 subpixels; therefore, by extracting the spectral information from the 16 subpixels contained within each sensor from the sliced spectral figure over the wavelength range from 400 nm to 900 nm, a three-dimensional gas information matrix composed of 16 vectors can be obtained.

The response of each sensor contained within the sensor array to the gas is inversely proportional to the distance from the sensor to the gas source. Therefore, by analyzing the intensity of the sensor responses at different positions within the array, we can then determine the spatial distribution of the gas across the entire sensor matrix area. In this study, the magnitude of the spectral change is quantified using the mean squared error (MSE), which is calculated as follows:

$$Response_{MSE}=\frac{1}{m\times n\times p}\sum_{i=1}^{m}\sum_{j=1}^{n}\sum_{k=1}^{p}{\left(X_{ijk}-Y_{ijk}\right)}^{2}$$

Here, *m* and *n* represent the column and row numbers of the gas information matrix, *p* represents the number of sliced spectral figures, and *X* and *Y* denote matrices that correspond to an arbitrary sensor and to the reference sensor located farthest away from the gas source, respectively. The responses are reflected in the heat map by color shading, as shown in Figure 2c. In summary, by comparing the changes in the spectra of any sensor with those of the matrix at the maximum distance from the gas source, the extent of the spectral variations across the sensors at different positions is quantified.

### 3.4. Distinguishing Multiple Gases with PCA Methods

In a previous work, we used PCA to extract the principal components of the differential spectra produced by the LSPR effect, thus enabling classification of the different gases [32,33]. In this study, we used the PCA results obtained from the previous work as a classification benchmark to aid in extracting the desired features from the gas information matrix corresponding to each sensor in the sensor array. The extracted features were then projected into the principal component space used to distinguish single gases, which allows gas classification to be performed in a multi-gas environment. Subsequently, we used heat maps to illustrate the spatial distributions of these gases. In the heat maps, each pixel represents a subpixel sensor, with the color indicating the classification determined via PCA, and the color intensity reflecting the magnitude of the MSE.

## 4. Results and Discussion

In this section, the morphology and the spectral features of the inkjet-printed LSPR gas sensors are reported. Based on this information, the fabricated inkjet-printed subpixel LSPR gas sensor array is used to detect four different gases. Additionally, the visualization performances for the evaporated gases will be compared based on gas type and concentration.

### 4.1. Surface Morphology of the Printed PET Substrate with Nanoparticles

**Figure 3:**
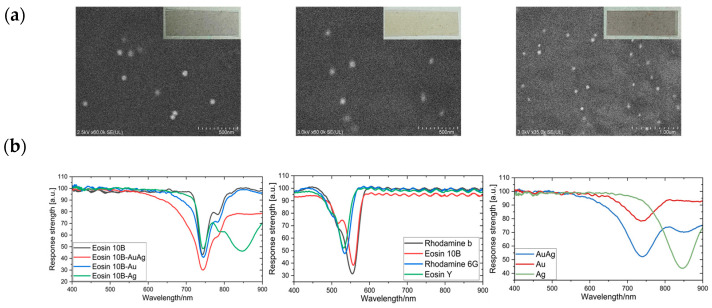
(**a**) Scanning electron microscope (SEM) images of the AuNPs (**left**), the AgNPs (**middle**), and both AuNPs and AgNPs (**right**) on the inkjet-printed LSPR sensor with the real printed pattern shown on the top right of each image in a different color. (**b**) Ultraviolet–visible (UV–Vis) spectra of Eosin with the different nanoparticles (**left**), a comparison of the different pigments (**middle**), and a comparison of the different nanoparticles (**right**).

Figure 3a shows scanning electron microscope images of the PET substrate surface with printed Au, Ag, and Au and Ag nanoparticles, which illustrate the surface nanoparticle distributions and morphologies. The surface element distributions were also detected by energy-dispersive X-ray spectroscopy (EDX) to confirm that the nanoparticles were placed on the surface of the PET substrate, as shown in Figure S4 and Tables S1–S3. PET substrates with different printed nanoparticles show different colors that depend on the component nanoparticles, thus inferring the existence of the LSPR phenomenon. The UV–visible (UV–Vis) spectra are shown in Figure 3b to enable comparison of the absorption peak for nanoparticles. The absorption peaks for the AuNPs and the AgNPs appear at 565 nm and 425 nm, respectively, which are the same peaks that were reported in previous research [38,39].

The spectra of the pigments are also shown in Figure 3b. Because the various fluorescent pigments have distinct spectral curves, the influence of these pigments on the LSPR effect of the nanoparticles varies. By combining the different fluorescent pigments with the nanoparticles, the absorption peaks can be altered correspondingly. For example, in Figure 3b, the absorption curves of the patterns formed by Eosin 10B in combination with the different nanoparticles are compared. The subpixels formed using the different combinations of fluorescent pigments and nanoparticles show unique spectral absorption characteristics. This enables acquisition of more comprehensive gas information in the subsequent experiments, thereby enhancing the gas identification accuracy.

### 4.2. Comparison of the LSPR Signals for Different Gases

**Figure 4:**
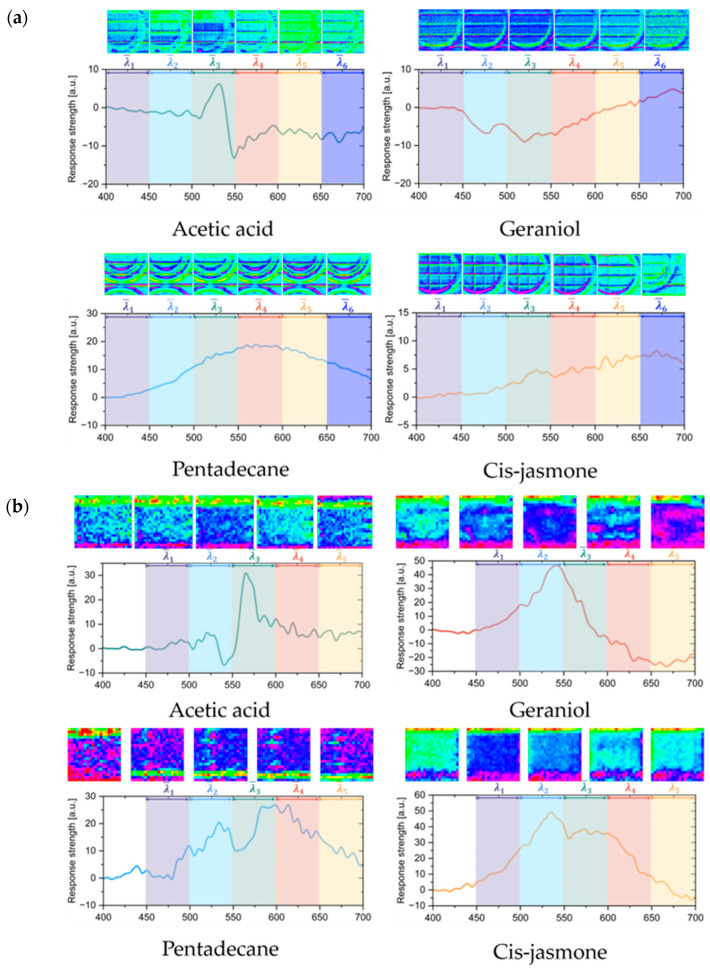
Differential spectra for 4 gases showing wavelength-dependent changes with respect to the average spectra over different ranges to highlight the differences. Acetic acid, geraniol, pentadecane, and jasmone are represented by the green, red, blue, and orange lines, respectively. (**a**) Average spectra obtained for a single subpixel sensor. (**b**) Average spectra obtained for another subpixel sensor.

The differential spectrum caused by the LSPR phenomenon can vary, depending on the gas environment. Figure 4a takes a single sensor as an example and shows the spectrum differences recorded at the different wavelengths. The average spectrum for all 16 subpixels in one sensor is calculated, and all four sensor responses to the different gases show differences that were not initially apparent. Here, the absorbance over the range from 400 nm to 700 nm is the average within the sensor area and is used to show the overall performance for the different gases. Based on this approach, the wavelength is sliced into six parts, and the average absorbance within each spectrum range is then calculated to generate the corresponding spectrum figure for the sensor, as shown below each spectrum curve. Although the average spectra for one sensor are not apparent, the differences among the subpixels can be found. For acetic acid, eosin Y and rhodamine B with nanoparticles show absorbance in the ranges from 450–500 nm and 500–550 nm, respectively, and the other subpixel shows less change, based on the sliced average spectrum. Therefore, the subpixel gas sensor shows a response to each of the different gases, but not all the subpixels in the sensor can show an apparent response to each gas.

By analyzing the responses of the same subpixel within the sensor to the different gases, we can observe distinct variations in the differential spectra generated by the different gas environments. As illustrated in Figure 4b, the subpixel composed of AuNPs, AgNPs, and eosin Y showed significant differences in its spectral response under the various gas conditions. For this subpixel, notable spectral changes occurred at 540 nm and 565 nm for acetic acid, with the strongest response being concentrated within this wavelength range, and this was reflected in the averaged spectral slices across the different ranges. The primary features of geraniol, pentadecane, and cis-jasmone were captured in the 500–600 nm, 500–650 nm, and 500–600 nm wavelength ranges, respectively, with each displaying distinct spectral trends. In summary, the subpixel gas sensor can gather gas information through all 16 of its subpixels. Because the spectral shifts caused by the LSPR effect are closely related to the type of gas, gathering differential spectra from all 16 subpixels enables more effective gas discrimination and visualization.

### 4.3. Visualization of Gas Spatial and Temporal Distributions

The total spectra were collected from the scale of an 11 × 12 gas sensor array for each detection process by the hyperspectral camera, and each sensor in the array contained 16 subpixels to allow different spectra to be acquired. As noted in Section 3.3, each sensor in the array can generate a unique gas information matrix. By considering the absorption peak distributions of the pigments and nanoparticles, the spectrum figure captured the ranges from 400 nm to 900 nm at 5 nm intervals, producing a total of 101 spectral slices, as illustrated in Figure 2c. Therefore, the gas information matrix size is 4 × 4 × 101, with its three dimensions representing the row and column positions of the subpixels within the matrix, and the spectral slices at the different wavelengths, respectively.

**Figure 5:**
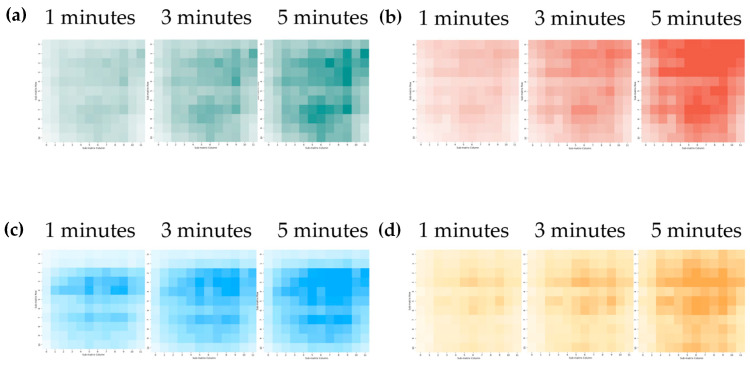
Visualization results for the gas spatial distribution and comparison of effects of exposure to a gas source for 1 min (**left**), 3 min (**middle**), and 5 min (**right**) for (**a**) acetic acid, (**b**) geraniol, (**c**) pentadecane, and (**d**) cis-jasmone. The X-axis and the Y-axis indicate the sensor location.

The impact of the gases on the LSPR effect is reflected in the changes observed in both the intensity and the positions of the absorption peaks. For the different gases, each subpixel within the sensor shows distinct absorption spectral changes caused by variations in the surface nanoparticles and the fluorescent pigments. In this study, the MSE among each of the elements in the gas information matrix is calculated based on the sensor located farthest away from the gas source in the initial state. This analysis was then used to generate the gas heat map shown in Figure 5, where each pixel in each heat map corresponds to an actual sensor in the array.

Here, the gas source is placed at the center of the field of vision. Therefore, the relevant pixel in the heat map shows darker colors when compared with the surrounding pixels. In addition, the color within the field of view becomes darker as time passes. One aspect that should be noted is that the color shade of the heatmap does not follow a diffusion pattern strictly from the center outward; instead, it shows a tendency to diffuse upward within the field of view. During the experiments, because the PET film becomes relatively soft even when fixed above the gas source, the distance between the film’s upper side and the gas source was slightly smaller than the distance from the lower side, thus causing better spectral data collection performance when comparing the upward and downward. This led to the upward diffusion trend observed during the gas dispersion process. Additionally, this characteristic indicates that the distance between the sensor and the gas source affects the sensor’s response. This relationship can be explored in future experiments by using more precise supports to fix the substrate and adjusting the distance between the substrate and the gas source.

**Figure 6:**
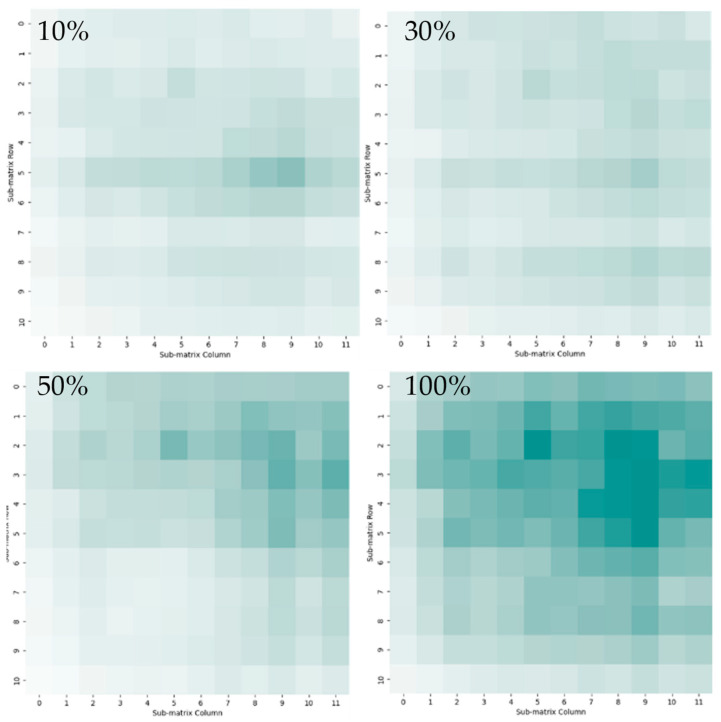
Acetic acid spatial distributions after 5 min with 10%, 30%, 50%, and 100% concentrations.

The concentration of the odor source solution can also affect the sensor detection and gas visualization results. In this case, acetic acid was used as an example substance at various concentrations (10%, 30%, 50%, 100%). The visualization results were compared after 5 min and the results of this comparison are shown in Figure 6. The color shades and darker areas varied proportionately with the gas source solution concentration.

### 4.4. Visualization and Identification of Multiple Gases

**Figure 7:**
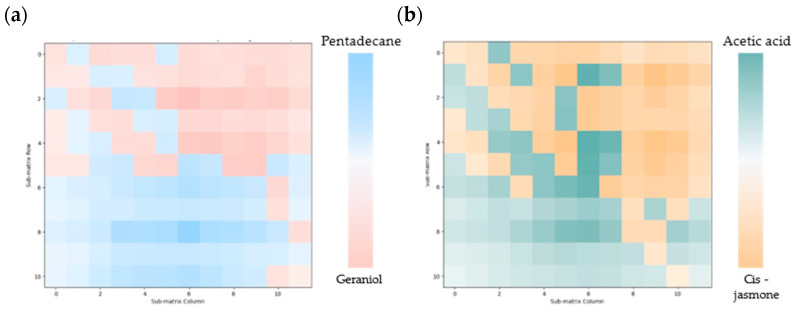
Visualization results for (**a**) pentadecane and geraniol, and (**b**) acetic acid and cis-jasmone based on a combination of the MSE and the PCA method.

The spatial distributions of the gases are shown in Figure 7. In this experiment, the four gas sources were divided into two groups and then placed in the lower left and upper right regions, respectively. The LSPR spectra were acquired from the differential images captured using the hyperspectral camera. In a previous work, a subpixel gas sensor was used to measure different gas types, and through PCA, the principal component vectors and the feature matrices corresponding to each gas information matrix were extracted, allowing successful classification to be achieved. Here, the average principal component vectors that were obtained when detecting the four gases separately were used.

To determine the primary gas compositions within the regions corresponding to each subpixel sensor, the PCA model was applied to reduce the dimensionality of the gas information matrix for each sensor, and the corresponding principal component vectors were then obtained. Comparison of the principal component vectors of each sensor’s gas information matrix with the principal component vectors of the four individual gases allowed the primary gas composition in the corresponding region to be identified. As shown in the results for each experiment, the main gas distribution areas were located near the corresponding gas sources. Furthermore, the spatial distributions of the different gases were influenced by their relative diffusion speeds, which led to variations in the distribution patterns between the two groups of gases.

The gas concentration information was visualized using the MSE. After the primary gas composition in each subpixel sensor’s range was identified through PCA, the differences between the sensor’s gas information matrix and the reference matrix were calculated. The limitations of this study are as follows: first, in areas in which the concentrations of two gases are similar, both gases can influence the LSPR spectra, and thus the MSE calculated after classification may reflect a mixture of the two gases. Second, the relatively large size of the subpixel gas sensor only allows for detection of gas differences over a 36 mm^2^ area. For applications that require higher precision when visualizing gas spatial distributions, the sensor size would need to be reduced to meet the detection accuracy requirements.

## 5. Conclusions

In conclusion, an inkjet-printed LSPR subpixel gas sensor array was constructed to perform gas spatial distribution visualization and to distinguish gases for acetic acid, geraniol, pentadecane and cis-jasmone odors. In addition, the distributions of gases based on their evaporation times and the relative percentages of the gas sources has been discussed. The inkjet-printed subpixel gas sensor, which combines AuNPs, AgNPs and fluorescent pigments, can gather spectrum information with its different subpixels to perform gas detection. In the gas detection and visualization experiment, the inkjet-printed LSPR sensor array was placed above the odor source at a height of 3 cm for gas adsorption. Then, the LSPR spectra of the gases were acquired by collecting the spectral figures for the sensor array using the hyperspectral camera. Differences exist between the spectra from the 16 subpixels in one sensor and those obtained from a subpixel with the same components in a different sensor in the sensor array. Here, the differences among the spectra obtained from the different subpixel patterns can describe the characteristics of the gases, while the differences among the sensors can describe the gas concentrations. For gas visualization, the gas information matrix was constructed using the spectral data from the 16 subpixels, and the MSE was calculated with respect to the sample sensor in the sensor array to obtain the concentration information of the gases and was then visualized using the concentration information plotted in the heat map. The PCA method was used to perform gas distinction for multi-gas detection applications. Here, the eigenmatrix and the eigenvector for distinguishing a single gas are used to identify the principal components in the feature information taken from the gas information matrix for each sensor in the sensor array. As a result, the four gas odor sources were identified and localized using a single inkjet-printed sensor array. This study presents a practical approach to the visualization of gas spatial distributions, with potential applications in identification and pinpointing the locations of odor sources across a range of fields. Additionally, the sensor fabrication method introduced in this study is simpler and more straightforward when compared with the fabrication methods used to form existing sensors, thus providing a new approach to the design of low-cost and convenient gas sensors and gas visualization systems.

In our future work, we intend to focus on the solutions to two primary challenges. First, we wish to improve the fabrication process of the inkjet-printed LSPR gas sensor array. When compared with other LSPR sensor fabrication methods, inkjet printing is more convenient and saves time, but the LSPR phenomenon is less apparent in the resulting sensors. We will also attempt to realize higher percentages of the gas components in one sensor area to make the gas visualization results more precise. In the real world, multiple gases can appear in the same area and form a gas mixture. Therefore, we will attempt to identify and visualize the components of these complex gas mixtures.

## Data Availability

The data are contained within the article and the Supplementary Materials.

## Supplementary Materials

The following supporting information can be downloaded at: https://www.mdpi.com/article/10.3390/s24206731/s1, Figure S1: The Size of pixel pattern and subpixel pattern; Figure S2: Part of the fabricated inkjet-printed subpixel sensor array. (a) Image of inkjet-printed pattern with nanoparticles. Yellow, dark purple, light purple, and colorless stripes can be observed in sequence, which are the patterns of the AgNPs, the AgNPs and AuNPs, and the AuNPs alone. (b) Pigments printed in sequence with different colors, and the scale of one subpixel sensor is indicated in blue; Figure S3: Image of the hyperspectral camera and the LSPR signal measuring system with the inkjet-printed subpixel sensor array; Figure S4: EDX results for a printed pattern. (a) Pattern for AuNPs. (b) Pattern for AgNPs. (c) Pattern for AgNPs and AuNPs; Figure S5: Results of gas identification among the four gases obtained with the PCA method from our former research. The pixel colors in the heat map for multiple gas sensing are based on these results; Table S1: EDX result for the AuNP pattern; Table S2: EDX result for the AgNP pattern; Table S3: EDX result for the AuNPs and AgNPs pattern.
