# Supplementary material for: Inkjet-Printed Localized Surface Plasmon Resonance Subpixel Gas Sensor Array for Enhanced Identification and Visualization of Gas Spatial Distributions from Multiple Odor Sources
Source: Sensors (Basel). 2024 Oct 19;24(20):6731. doi: 10.3390/s24206731 (PMC11511064; doi:10.3390/s24206731)
Supplement: Supplementary file 1 [file sensors-24-06731-s001.zip › sensors-3265344-supplementary.pdf]

Innovative Inkjet Printed LSPR Subpixel Gas Sensor Array for Enhanced Identification and Visualization of Gas Spatial Distributions from Multiple Odor Sources

Tianshu Jiang[1], Hao Guo[1], Lingpu Ge[1], Fumihiro Sassa[1], Kenshi Hayashi[1]

Experimental method

Ink preparation

Nanoparticle ink was prepared by adding PVP K30 1.5mg and triton-100 1.5 μl mixed with Au or Ag nanoparticle solution without any treatment. After that, the nanoparticle ink will be loaded into the printer cartridges (Epson PX-105, Japan). When the pattern of the corresponding ink needs to be printed, the ratio of the two inks can be achieved by controlling the basic color percentage. Here, the surface tension is 33.84 mN/m, and the viscosity is 1.34 cP to realize stable printing results. All nanoparticle patterns were printed 30 times.

Rhodamine B, Rhodamine 6G, Eosin Y, and Eosin 10B were dissolved in 50% ethanol to form 1μM solutions. Pigment patterns were printed after the nanoparticle printing, with each pattern applied three times to achieve the desired nanoparticle-pigment composition. Here, 4.32×10^{-5} ml ink is placed in 1.2×1.2 mm^2 square from the pattern for nanoparticles and pigments in one-time printing. All pigment patterns were printed 3 times. The PET substrate was dried in an oven at 60 ℃ 4 hours later.

Supplementary figures and tables Figures

Figures

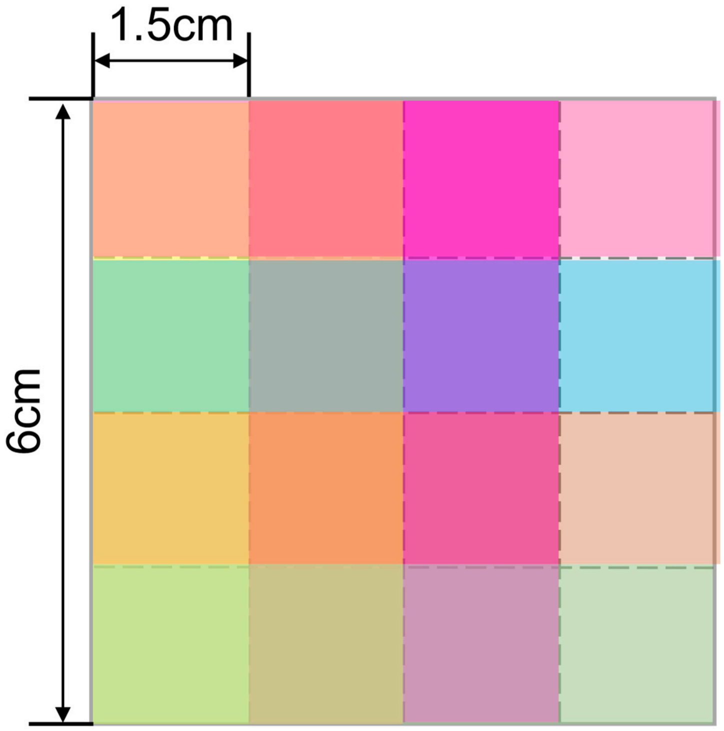

Figure S1. The Size of pixel pattern and subpixel pattern

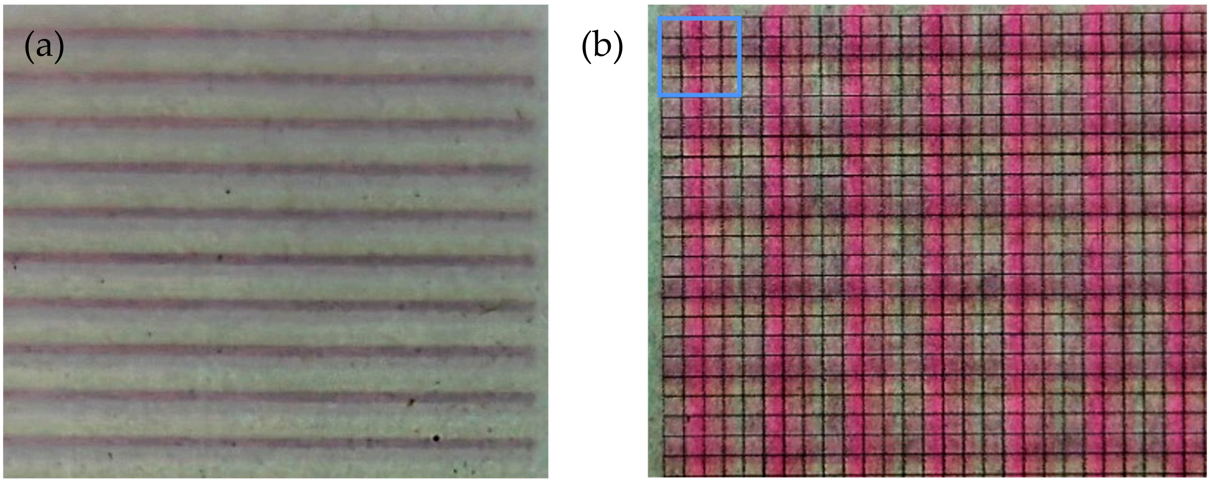

Figure S2. Part of fabricated inkjet printed subpixel sensor array. (a) The image inkjet printed pattern with nanoparticles. Yellow, dark purple, light purple and colorless stripes can be seen in

sequence, which are AgNPs, AgNPs & AuNPs and AuNPs pattern. (b) Pigments are printed in sequence with different color, and the scale of one subpixel sensor is signed in blue.

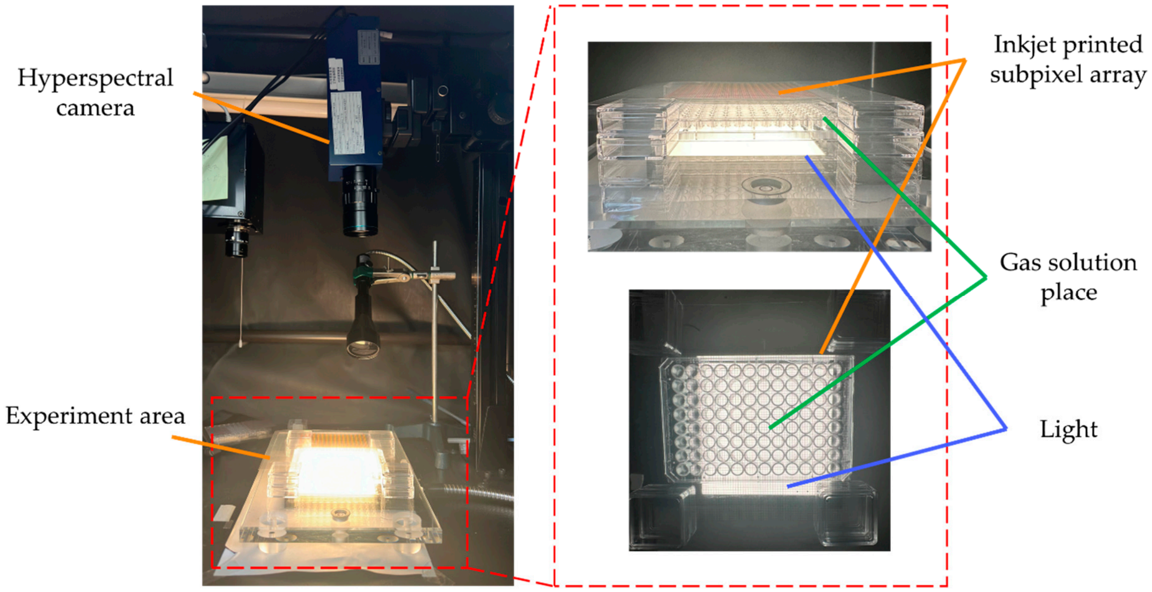

Figure S3. The image of the hyperspectral camera and LSPR signal measuring system with inkjet printed subpixel sensor array.

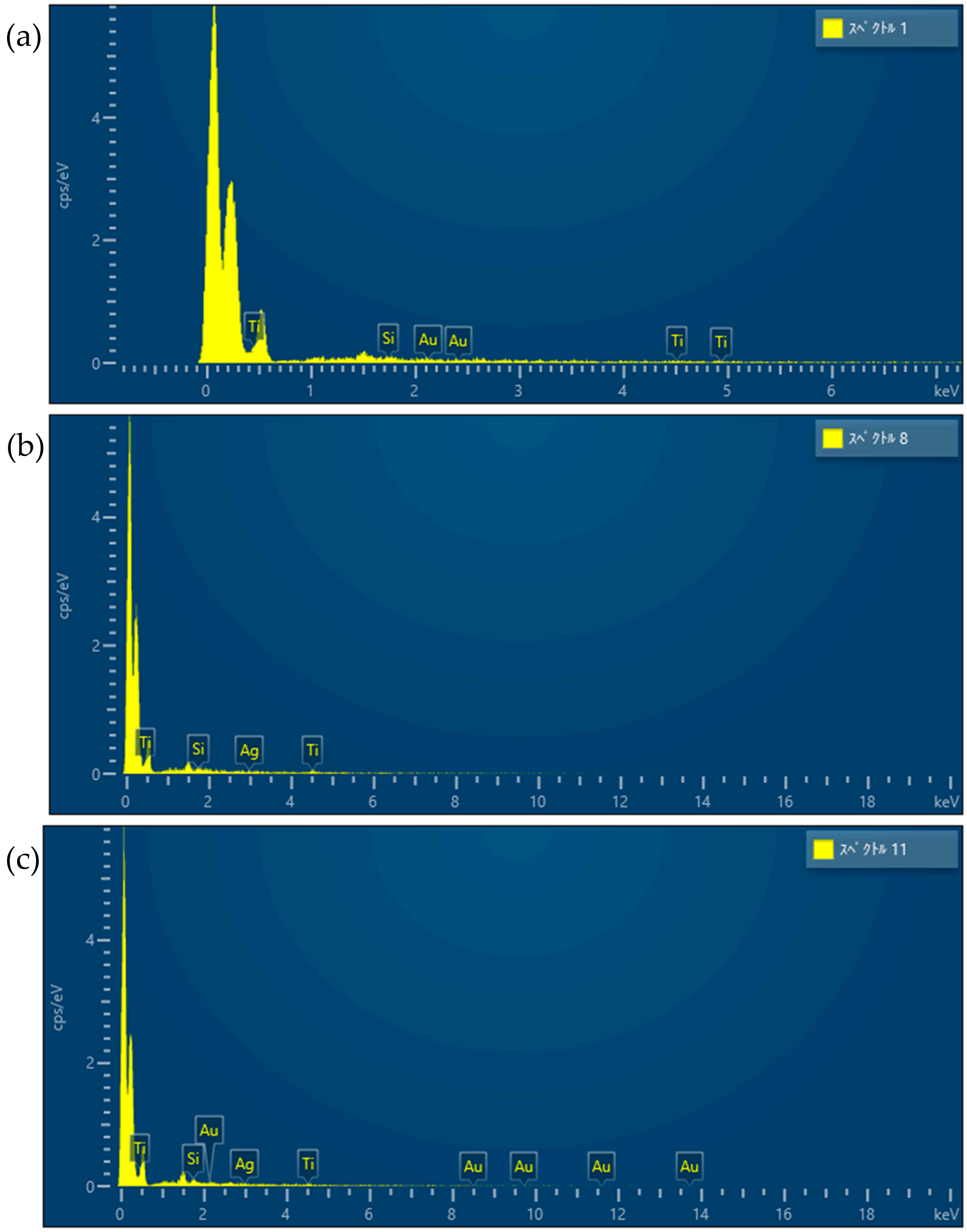

Figure S4. The EDX result of printed pattern. (a) Pattern of AuNPs. (b)Pattern of AgNPs. (c) Pattern of AgNPs & AuNPs.

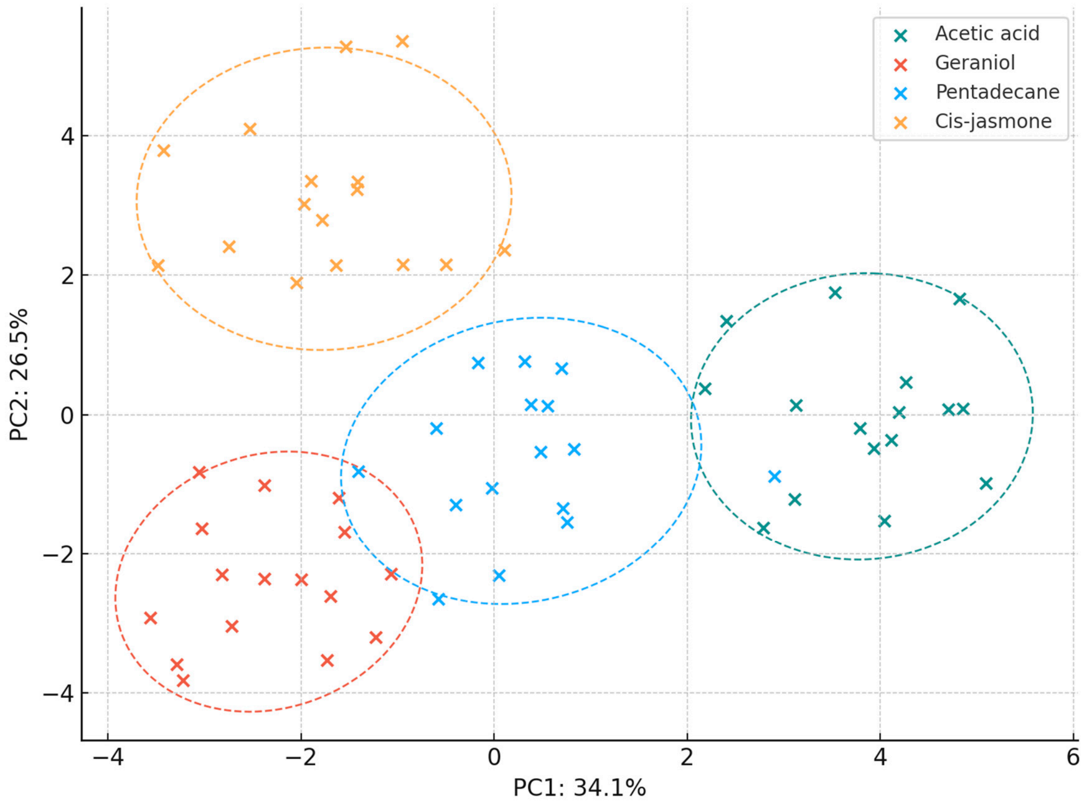

Figure S5. The result of gas identification among the 4 gases with PCA method with our former research. Pixel color in heat map for multiple gas sensing is based on this result.

Reference

30. Jiang, T. et al. Integrated Subpixel-Patterned LSPR Gas Sensor via Inkjet Printing of Au/Ag Nanoparticles and Pigments for Multigas Detection. IEEE Sens. Lett. 8, 1–4 (2024).

31. Jiang, T. et al. Subpixel Patterned LSPR Gas Sensor Array With Using Inkjet Printing Au/Ag Nanoparticle to Enhance the Selectivity. IEEE Sens. Lett. 7, 1–4 (2023).

Tables

Elements	Line type	Mass %	Atomic number density%	Oxide	Oxide Mass%
Si	K Series	22.89	21.72	SiO2	48.98
Au	M Series	20.67	2.80	Au2O3	23.19
Ti	K Series	16.69	9.28	TiO2	27.84
O	K Series	39.75	66.20		
Total		100.00	100.00		100.00

Table S1. The EDX result of AuNPs pattern.

Elements	Line type	Mass %	Atomic number density%	Oxide	Oxide Mass%
Si	K Series	16.49	15.55	SiO2	35.28
Ag	L Series	15.71	3.86	Ag2O	16.87
Ti	K Series	28.69	15.86	TiO2	47.85
O	K Series	39.11	64.74		
Total		100.00	100.00		100.00

Table S2. The EDX result of AgNPs pattern.

Elements	Line type	Mass %	Atomic number density%	Oxide	Oxide Mass%
Si	K Series	21.19	20.40	SiO2	45.33
Au	M Series	13.25	1.82	Au2O3	14.86
Ag	L Series	8.64	2.17	Ag2O	9.29
Ti	K Series	18.30	10.33	TiO2	30.52
O	K Series	38.62	65.28		
Total		100.00	100.00		100.00

Table S3. The EDX result of AuNPs & AgNPs pattern.

Reference

30. Jiang, T. et al. Integrated Subpixel-Patterned LSPR Gas Sensor via Inkjet Printing of Au/Ag Nanoparticles and Pigments for Multigas Detection. IEEE Sens. Lett. 8, 1–4 (2024).

31. Jiang, T. et al. Subpixel Patterned LSPR Gas Sensor Array With Using Inkjet Printing Au/Ag Nanoparticle to Enhance the Selectivity. IEEE Sens. Lett. 7, 1–4 (2023).
